# Rural student mobility and the spatial politics of university access in Nigeria

**DOI:** 10.1007/s10734-025-01592-z

**Published:** 2025-12-01

**Authors:** Yusuf Damilola Olaniyan

**Affiliations:** Department of Education, University of Bath, Bath, UK

**Keywords:** Higher education, Rurality, University access, Spatial inequalities, Choice of university, Decoloniality, Nigeria, Postcolonial

## Abstract

Despite the post-independence reforms aimed at addressing educational inequalities in university access in Nigeria, figures for rural students have not improved. While policy frameworks such as the quota system and centralised examinations are routinely celebrated as instruments of fairness, they have not translated into substantive equity for rural students. This research provides a novel understanding and explanation of how rural Nigerian students choose and access university education. It drew on 45 in-depth interviews across three federal universities in Nigeria. The study found two interrelated mobility logics that influenced rural students’ choice of university: staying close to home (primary sorting), shared identity and the boundaries of belonging (secondary sorting). It also found institutional barriers influencing access: Joint Admissions and Matriculation Board (JAMB) and post-JAMB examination. Guided by a postcolonial lens, the analysis draws on Mamdani’s theory of decentralised despotism to show how colonial governance structures continue to shape Nigeria’s internal geographies of access. This is complemented by Bourdieu’s concept of habitus, used to understand how spatial and symbolic inequalities are embodied in students’ educational choices. The study argues that access should be understood not as a single point of entry but as a contested and layered journey. It calls for policies that move beyond increasing enrolment to address the invisible borders, spatial hierarchies, and infrastructural gaps that shape who moves, where they go, and under what conditions.

## Introduction

In postcolonial Nigeria, who accesses university, and under what conditions is shaped by geography, history, and inherited structures of inequality (Olaniyan, 2025; Olaniyan & Donnelly, 2025). Official metrics of inclusion tend to focus on aggregate enrolment and centralised admission thresholds, conspicuously through the Unified Tertiary Matriculation Examination (UTME) administered by the Joint Admissions and Matriculation Board (JAMB). JAMB is Nigeria’s centralised admission body that regulates entry into all public and many private universities. They also determine admission quotas, and oversee Direct Entry (DE) pathways, which allow candidates with diploma or A-level qualifications to enter at the second-year level (Olaniyan, 2025). In addition, many universities administer “Post-JAMB” screening, typically in the form of aptitude tests or interviews, to further evaluate applicants beyond their UTME results (Olaniyan., 2025). While public universities remain largely subsidised, students are still responsible for tuition and various service charges. Moreover, many state-owned universities implement indigenisation policies that reduce tuition fees or preferential admission to candidates from the host state. While enrolment has grown from 210 students in 1948 to over 2.1 million by 2019 (NUC, 2019), the student participation rate remains below 15% (Okebukola, 2018). While this expansion, as Isiaka (2024) argues, “might signal a system transitioning from an elitist to a mass (Trow, 1973) or high participation system (Marginson, 2016)” (p. 1), it simultaneously conceals persistent exclusions, particularly for students from rural and underserved regions. For example, at the levels preceding university, non-completion is overwhelmingly rural: at junior secondary, 79% versus 21% urban; at senior secondary, 78% versus 22% (UNICEF, 2023).

Across the Global South, colonial administrative divisions, infrastructural disparity, and cultural heterogeneity have shaped spatialised patterns of educational investment (Okoye & Pongou, 2014). In Nigeria, this inheritance intersects with rapid urbanisation (World Bank, 2022) and intra-national inequality, which Horner et al. (2018) describe as “territorial proximity”, now becoming a defining axis of global disparity. Nevertheless, most rural-origin youth who migrate to cities for education and employment are forced to navigate these transitions without institutional recognition of rurality as a structurally significant barrier (Okhankhuele & Opafunso, 2015).

Drawing on forty-five in-depth interviews across three federal universities in South West, North West, and North Central Nigeria, this study interrogates how rural origin mediates the routes through which young people access university. It reveals how spatial constraints, inherited fears, and regional belonging shape student decision-making in ways that disrupt dominant narratives of rational, individual ‘choice’. Rather than navigating a neutral field of institutional options, rural students operate within bounded geographies of possibility, where proximity, identity, and perceived risk frame what is considered achievable or survivable.

To account for these patterned logics, the analysis develops a conceptual framework that builds on Boudon’s (1974) distinction between primary and secondary effects in educational stratification, while extending it beyond a narrow class lens. In the Nigerian context, where education has historically served as a tool of both colonial governance and postcolonial differentiation (Mamdani, 1996; Olaniyan, 2025), spatial and cultural belonging are as consequential as socioeconomic status. What emerges is a typology of access that foregrounds how students filter, negotiate, and resist the conditions of their mobility, logics that are shaped as much by history and region as by aspiration.

It is argued that these trajectories are not expressions of preference alone, but responses to the infrastructural and affective architecture of the Nigerian state. The 1914 colonial amalgamation that joined Northern and Southern Nigeria under administratively distinct systems continues to shape educational geography (Okoye & Pongou, 2014). As Mamdani (1996) theorises, decentralised despotism in postcolonial Africa entrenches regionally differentiated authority structures, making cross-zonal movement neither ideologically neutral nor institutionally seamless. What is often framed as choice is, in practice, a historically sedimented response to structural risk.

This article, therefore, centres the voices and strategies of rural students to contribute to critical debates on HE access and spatial justice. It challenges the technocratic orthodoxy that equates access with admission and calls for a reconceptualisation that foregrounds movement, belonging, and constraint. In doing so, if access is as much about the places students aspire to as the places they feel unable to reach, then university participation must be understood not just by where students go, but by where they learn they cannot.

## Spatial inequalities and university access in Nigeria: literature review

Contemporary debates on higher education (HE) access in sub-Saharan Africa have often centred on the twin pressures of expansion and equity (Agbaire, 2018; Isiaka, 2024). National governments have embraced HE as a pathway to economic growth, technological advancement, and global competitiveness (Oketch, 2016). However, scholars caution that the massification of HE, while ostensibly inclusive, risks deepening existing inequalities unless it is underpinned by structural reforms attentive to the lived conditions of marginalised populations (Agbaire, 2018; Isiaka, 2024). In the Nigerian context, where educational development has been historically fractured by colonial legacies, religious diversity, and regional governance disparities (Agbaire, 2018; Isiaka, 2024; Olaniyan, 2025), rural students remain caught in a landscape where access is shaped by infrastructural reach and by questions of recognition, of who is imagined within the national educational project, and on what terms.

Much of the literature on university access in Nigeria remains anchored in quantitative indicators and positional arguments, around enrolment, gender disparities, and financial constraints (Esomonu & Adirika, 2012; Ogunode & Musa, 2020; Emmanuel et al., 2015). While such studies have provided valuable insights into systemic inequities, they often position rurality as a static category, as a demographic variable to be measured rather than a lived and contested positionality within the Nigerian educational field. More recent qualitative and spatially attuned scholarship has moved towards viewing access as a dynamic process, shaped by infrastructural marginality, social location, and cultural legibility (Wilson-Strydom, 2015; Temple, 2009; Morley & Lugg, 2009). These studies offer a more textured reading of access, not as a singular threshold, marked by admission, but as a layered, uneven, and affectively charged journey shaped by institutional architectures and regional hierarchies.

However, what remains underexplored are the material and everyday barriers through which spatial inequality is reproduced. Rural students often face long travel distances to universities, compounded by high transport and relocation costs (Fagbemide, 2025). Many must secure off-campus housing in urban centres, which raises living costs beyond tuition and fees. Limited internet connectivity, erratic electricity supply, and the high cost of digital devices also constrain rural students’ ability to prepare for the Unified Tertiary Matriculation Examination (UTME) and access online admission portals (Olanrewaju et al., 2021; Okocha & Dogo, 2023). Insecurity during rural–urban travel, particularly in Northern Nigeria, adds another layer of exclusion, as families restrict mobility out of safety concerns (Okocha & Dogo, 2023). These structural conditions illustrate that access is not only about securing admission but about sustaining the possibility of participation across space.

Similarly, existing studies and debates have explored educational inequality in Nigeria through the lens of class, gender, disability, policy, and indigeneity (e.g., Agbaire, 2018; Isiaka, 2024; Okebukola, 2018; Olaniyan, 2025). However, relatively little attention has been paid to how spatial politics shape rural students’ trajectories in accessing university education. Moreover, most quantitative analyses of access fail to capture the narrative decisions that underpin mobility choices. This study contributes to the literature by focusing on the qualitative narratives to entry faced by rural students and how their movements, refusals, and institutional navigations reflect broader sociohistorical logics that are often invisible in policy discourse. It extends conversations on meritocracy and inclusion by lacing the spatial dimensions of aspiration and exclusion in a postcolonial context.

Furthermore, a growing body of work, particularly within African and postcolonial contexts, has also interrogated how colonial histories continue to contour spatial patterns of opportunity. Mamdani’s (1996) formulation of decentralised despotism remains instructive for understanding how indirect colonial rule bifurcated territories into centralised urban cores and subordinated rural peripheries. In Nigeria, this legacy produced an enduring duality: missionary-led educational expansion in the South and a more conservative, religiously embedded educational model in the North (Okoye & Pongou, 2014). The uneven spatialisation of education under colonial rule has persisted and re-inscribed through postcolonial policies that, while ostensibly national in scope, often reinforce historical geographies of neglect (Olaniyan, 2025). For example, Van de Walle (2009) shows how state investments in tertiary institutions have disproportionately favoured urban or politically strategic regions, leaving rural areas undersupplied and symbolically peripheral.

Such spatial asymmetries are not merely logistical since they also shape the affective and epistemic logic of access. In their work on South Africa, Walker and Mkwananzi (2015) note that students from marginalised geographies experience physical and symbolic distance from institutions and from the cultures and expectations of university life. Uleanya (2022) also researched the learning challenges of rural students in Nigeria and South Africa, but this study focused on academic outcomes and did not fully unpack the intersection of access and mobility. Even in the Global North, Farrugia and Ravn (2022), in their book, argued that influential sociological thoughts from the Global North about social life are presented from the vantage point of the urban or modernity, and spaces beyond the city are considered pre-modern and underrepresented. Similarly, Brown and Donnelly (2023), drawing from international education research from the global South and North, recount the need for research advancing social and epistemic justice and educational outcomes from the spatial reality of the students.

### Theoretical logic

This study is grounded in a relational understanding of access that resists framing university admission as the predictable outcome of individual merit or institutional policy. Instead, it aligns with anti-deficit frameworks (Harper, 2010; Yosso, 2005), which shift focus away from presumed student shortcomings and toward the social, spatial, and political contexts that shape opportunity. Access is conceptualised as a spatially encoded, historically sedimented, and socially navigated process, one that cannot be abstracted from the logics of colonial governance, infrastructural asymmetry, or everyday negotiations with risk and legitimacy.

To capture these dynamics, this research is situated within a postcolonial framework, which interrogates how colonial legacies continue to shape contemporary social, political, and educational structures in newly independent states (Bhambra, 2014). Postcolonial theory draws attention to how power relations, institutional forms, and ways of knowing that originated during colonial rule are often re-inscribed in post-independence contexts, sometimes in more subtle or technocratic forms (Bhambra, 2014). It is this ongoing entanglement of past and present that provides the critical lens for understanding spatial and institutional exclusions in HE. Within this framework, this study was shaped by two theoretical commitments. First is Mamdani’s (1996) theory of decentralised despotism, which frames postcolonial states not as fully integrated national spaces but as territorially fragmented regimes, where colonial logics of indirect rule continue to organise access to resources, legitimacy, and institutional authority. In the Nigerian case, the dual legacy of British indirect rule in the North and missionary-led education in the South has produced persistent regional disparities in educational infrastructure, epistemic legitimacy, and institutional culture. These disparities do not merely sit in the background of university access, they structure the very maps students read when imagining where they can go, where they belong, and where they may not be safe. Mamdani’s work provides a critical vocabulary for understanding students’ decisions to avoid certain regions, select proximate institutions, or perceive some universities as culturally or politically inaccessible.

This framework is particularly useful in understanding Nigerian HE, where quota-based admissions, catchment area policies, and regional balancing reproduce colonial-era logics of segmentation (Olaniyan, 2025). ‘Despotism’ emerges in the unaccountable, centralised control over who gets access, often without transparency, while ‘decentralisation’ is visible in the regionalisation of opportunity through unequal institutional development. In effect, as shown in the findings of this work, students are still navigating a HE system stratified by colonial and postcolonial mappings of territory, identity, and legitimacy. Besides, the proliferation of federal universities across geopolitical zones performs a logic of inclusion, yet reinforces spatial fixity, with students internalising inherited maps of legitimacy and safety. In this sense, despotism persists in how spatial and institutional power is exercised, selectively and unequally, under the guise of unity and representation.

Second, the study draws on Bourdieu’s (1977) concept of habitus, reworked here to attend to the spatial and postcolonial contexts of educational decision-making. Rather than a static class-based disposition, habitus is understood as a dynamic orientation formed through cumulative exposure to regionally coded resources, familial advice, religious affiliation, and social scripts. It governs how risk is felt, how aspiration is tempered, and how ‘reasonable’ choices are constructed. For rural students, choosing a university is not merely an exercise in preference; it is a habitus-mediated response to histories of exclusion, collective memory, and infrastructural absence. The instinct to stay close to home, to choose the familiar over the prestigious, or to avoid regions coded as unsafe, emerges not from inertia but from an embodied logic of survival.

These frameworks directly shaped how the data were interpreted. Mamdani helped illuminate the historical structuring of spatial risk, while Bourdieu enabled attention to the affective and relational reasoning embedded in students’ narratives.

## Methodology

This study is situated within a qualitative, interpretivist tradition that understands knowledge as co-produced, partial, and embedded in social context. An in-depth narrative approach was adopted to explore how rural students encounter and interpret university access, not simply as a policy event, but as a situated experience shaped by geography, memory, and identity. Conventional indicators, such as enrollment rates and test scores, offer little insight into how access is negotiated, feared, or recalibrated through everyday life. The objective, therefore, was to develop an approach sensitive not just to what was said, but to how it was told: to the silences, hesitations, repetitions, and narrative shifts that give texture to lived experience.

Forty-five (45) undergraduate students were interviewed across three federal universities located in the South West, North West, and North Central regions of Nigeria. See Table 1. The three federal universities were chosen to reflect geographic, cultural, and institutional diversity across Nigeria’s zones. The Southwest university is older and nationally prestigious, while the Northwest and Northcentral universities draw more regional students. They were selected for accessibility to rural populations and proximity to catchment areas, enabling exploration of spatial logics across contexts.

**Table 1:** Distribution of student participants by region, university type, gender, and year of study

Region	University Type	Total	Male	Female	Year 1	Year 2	Year 3	Final Year
South West	Federal	**15**	8	7	4	3	5	3
North West	Federal	**15**	9	6	3	4	4	4
North Central	Federal	**15**	7	8	5	3	3	4

Table 1 presents the distribution of participants by region, university type, gender, and year of study. In the findings section, participants are referred to using pseudonyms accompanied by brief identifiers (e.g. pseudonym, gender, and region) to preserve anonymity while allowing readers to follow individual narratives and locate them within the broader socio-spatial context.Participants were purposively selected based on rural origin, with attention to variation in gender, admission route (JAMB or Direct Entry), and year of study. Gender was included as a key criterion to reflect broader national patterns of gendered participation in Nigerian HE and to explore how intersecting identities shape access.

Interviews were conducted in English and in-person, and later transcribed with attention to cultural and linguistic nuance. Participants were identified through a combination of purposive and snowball sampling. Initial contacts were made through student representatives, university course groups, and faculty referrals. From these entry points, other rural-origin students were referred by peers. I also work with a local NGO (Five Cowries Arts Education Initiatives) that works directly with students in rural communities in Northwest Nigeria. The decision to purposively select 15 participants from each university was made because of the desire to achieve institutional balance and capture both cross-site comparison and thematic depth. Each interview lasted between 20 and 40 min, was audio-recorded, transcribed, pseudonymised, and treated as a narrative account rather than a set of responses.

**Fig. 1:**
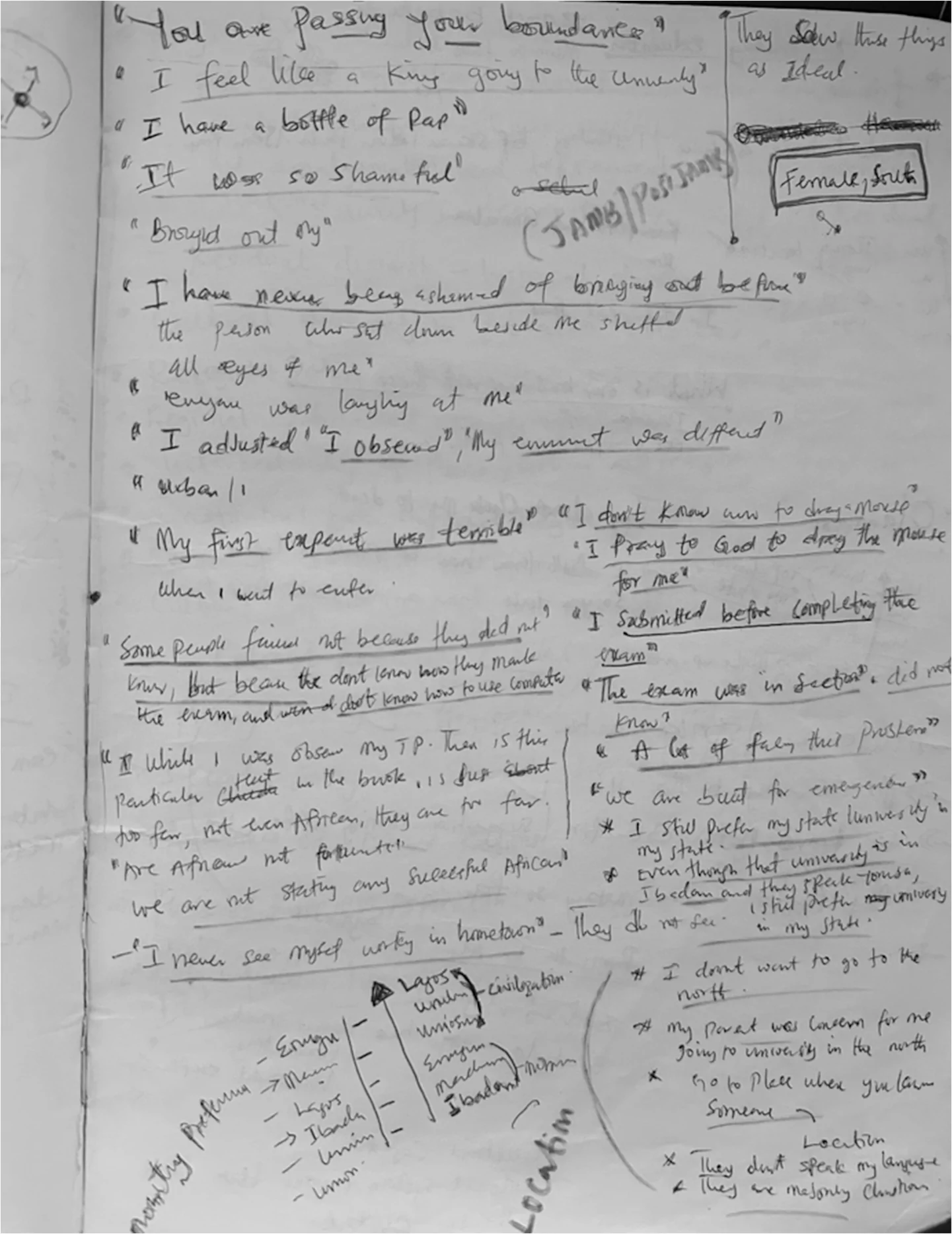
Page from the researcher’s reflexive journal documenting initial impressions, emotional tone, and contextual observations following interviews

Alongside the recorded interviews, I maintained a reflective journal in which I noted initial impressions, emotional tone, and contextual observations immediately after each conversation. These reflexive notes helped me begin identifying emerging patterns and supported a constant comparative approach during analysis. See Fig. 1 below, a page from my reflexive journal.

The analysis was guided by two complementary frameworks. The first was the Habitus Listening Guide (Naveed & Arnot, 2019), which offered a systematic method for attending to the layered nature of rural student narratives. Rather than treating each participant’s account as a unified voice, the guide encouraged listening across different registers: social positioning, intergenerational scripts, religious logics, and spatial imaginaries. In practice, this meant reading transcripts repeatedly, noting where participants spoke with confidence or caution, and identifying shifts in tone when narrating decisions about location, safety, or institutional reputation. I listened for discontinuities between stated intentions and lived decisions, tracing how habitus, understood as a historically formed and spatially mediated disposition, surfaced in everyday reasoning. For example, one participant initially spoke of wanting to attend a prestigious university in the South but later described choosing a nearby institution because “my uncle said, ‘Go to where you know people.’” The shift revealed how familial caution and spatial familiarity overrode individual aspiration, an expression of habitus operating beneath articulated desire.

Alongside this, I employed reflexive thematic analysis (Braun & Clarke, 2019), which enabled me to identify patterned meaning across the dataset without imposing fixed categories. Themes were constructed inductively through iterative engagement with the transcripts. Rather than coding in a mechanistic way, I approached each transcript as a dialogic space, interpreting accounts in relation to both their internal logics and the broader structural conditions they evoked. Coding commenced after transcription and was conducted manually. However, the insights I gained from reflexive journaling (Fig. 1) during data collection meant that analysis had already begun in a more fluid, observational form. This early engagement helped me refine and revisit emerging ideas as I progressed, maintaining an ongoing dialogue between field notes and formal transcripts.

Following Braun and Clarke’s reflexive model, I acknowledged that themes are not discovered but crafted through the interpretive relationship between researcher and text. They reflect my own intellectual, emotional, and cultural positioning. As someone born and educated in Nigeria but trained in Euro-Western academic institutions for a master’s and a PhD, I share a rural background with my participants and have experienced the spatial and institutional transitions they describe. However, I do not present myself as a neutral observer. Rather than being “ambivalent,” my position reflects what Narayan (1993) describes as multiplex subjectivity, a fluid and layered positionality shaped by personal experience, academic training, and institutional affiliation. This duality shaped how I was perceived during interviews: at times as an insider who understood rural realities, and at other times as an academic from a UK university. Acknowledging this complexity allowed me to navigate both empathy and distance, recognising how my standpoint influenced the co-construction of meaning throughout the research.

The combination of the Habitus Listening Guide and reflexive thematic analysis allowed for a balance between conceptual rigour and narrative sensitivity. While the listening guide attuned me to the relational and cultural grammars within each account, thematic analysis helped distil recurring logics, such as the pull of geographic proximity, the avoidance of perceived risk, and the emotional weight of aspiration. These themes did not emerge as static categories but as provisional frameworks for organising experience, grounded in the fragments, tensions, and strategies students mobilised in navigating access.

Ethical approval for this study was obtained from the University of Bath, as well as from the relevant ethics committee of the participating university where the research was conducted. All participants were fully informed about the purpose and procedures of the study and gave their voluntary, informed consent prior to participation. Confidentiality and anonymity were assured, and participants were informed of their right to withdraw from the study at any stage without consequence.

The choice to prioritise voice over numerical abstraction was both a methodological and political decision. Following Naveed & Arnot’s (2019) emphasis on hearing the “harmonics of voice,” I treated rural narratives not as deficits but as archives of insight, resistant to simplification and rich in generative complexity. In doing so, this research affirms that rural students are not marginal figures in need of rescue, but epistemic agents navigating a stratified educational space.

## Results

This section presents two interrelated logics shaping rural students’ university choices, primary sorting and secondary sorting, alongside institutional barriers such as JAMB and Post-JAMB. Emerging from reflexive thematic analysis, these themes show how geography, identity, and institutional systems overlap in structuring opportunity. Rather than fixed stages, they represent layered and relational forces through which rural students made sense of access to higher education.

### Part 1: patterns of mobility and university choice

#### Primary sorting: staying close to home

This first layer of mobility decision-making, Primary Sorting, captures how location functions as a dominant logic in rural students’ navigation of university access. Unlike Boudon’s (1974) primary effects, which are concerned with academic performance shaped by social origin, Primary Sorting in this context refers to the instinctive filtering of university options based on spatial proximity and the reassurance of what is already known. For many participants, location was not simply a factor; it was the organising principle through which aspirations, opportunities, and anxieties were negotiated. This was true even in cases where the location of other universities shared students’ language, ethnicity, or religious background; what mattered most was not identity affinity but the territorial logic of proximity and state-based belonging.

Participants repeatedly ranked proximity as the most influential criterion in their choice of university. One student (David, Male, Southwest) stated,

*"If I am to rank number one, number two, number three, or number four, that would be the University of Asa (pseudonym). The reason is that…in my own choice, it is about the location."*

Another added,

*“I still prefer universities in my state… the University of Basa (pseudonym) is in my state. So*,* I should support my state*.”(Funmi, Female, North Central).

In such expressions, what surfaces is a calibrated preference for the familiar, particularly when students or their families anticipate vulnerability in less predictable environments.

The logic of Primary Sorting was especially visible in how students described the idea of movement across zones. For some, unfamiliar locations were not evaluated in terms of institutional quality but dismissed outright due to their spatial distance and the perceived absence of familiar structures. A student (Amaka, Female, South South) explained her decision to choose the University of Asa over Basa by noting,

*"I have never been to that place… everything will just be strange. But here in Asa, I already have people that will guide me through like everything."*

In this sense, proximity conferred not only logistical convenience but also a safeguard against perceived exposure and disorder.

Cost and transportation were also central to these decisions. In a context where travel infrastructure is unreliable and students often shoulder the burden of navigating access alone, remaining nearby becomes a way of reducing both material and emotional costs.

*“At least if I am going to Ibadan now*,*” one student said*,* “I know that if I have like ₦5000 I will be in my house. But that Enugu*,* Kano you are saying*,* if you don’t have anything like ₦40*,*000 you can’t go.”* (Bade, Male, Southwest).

This comment illustrates how geographic reach is shaped by economic margin. Even when other regions offer institutional opportunities, they fall outside the range of consideration because they are financially or logistically inaccessible.

Parental influence was also significant in reinforcing this pattern. Several participants recounted how family members expressed concern over students moving too far from home. One (Seyi, Female, Northcentral) noted,

*"My parents said, ‘Who will take care of you there?’ So I had to look for something nearby."*

Another (Dada, Female, Northwest) recalled being advised by a relative,

*"Go to a place where you have people. If anything happens, who will help you in another region?’ That stayed in my mind."*

These remarks point to a broader ethic of protective reasoning that favours predictability over prestige. Decision-making is rarely treated as an individual act; rather, it emerges from collective strategies of containment aimed at reducing perceived exposure to harm.

Students also clarified that even when cultural affinity existed, through shared language or religion, it did not outweigh the imperative to remain within their own state. As one participant explained,

*“I am from southwest and we speak Yoruba*,* but I will choose university in my state before any other Yoruba-speaking state*,* you get…like Ondo now. I remember when I tried admission into university in my state twice and did not get it*,* some people advise me to apply to a university in Ekiti since it is less competitive compared to the university in my state…somebody even said they know someone that can help with admission there*,* and you know they speak Yoruba there too*,* but something in me just don’t want to go there.”* (David, Male, Southwest).

Another added,

*“No*,* for me*,* I don’t consider if they speak my language there or have a majority of people practising my religion first*,* I still chose university in my state first…you know this catchment policy thing can also favour me if I choose university in my state.”* (Bade, Male, Southwest).

In many cases, the decision to remain local is less a preference than a pattern shaped by observation. Students spoke of choosing institutions that others in their community had attended, thereby reproducing a form of mobility that is pre-scripted by local precedent. As one (Sade, Female, Southwest) explained,

*"Everyone in my village who goes to university, they go to the same schools. It’s like a road we all take."*

This recursive logic reveals how familiarity operates not only at the level of space but also as a form of intergenerational knowledge transmission. When older siblings or neighbours have already navigated a particular route, it becomes legible and trusted.

Even in instances where students initially considered moving further afield, the prospect often gave way to a return to the familiar. One participant (Femi, Male, North Central) confessed,

*"I always said I wanted to leave, to experience something different. But with these options, I’d still pick somewhere in my region. It just makes sense."*

The imagined risks of distance frequently outweigh the potential gains, especially in contexts where institutional support for transitioning students is limited or uneven.

Finally, state-level catchment policies, while designed to address regional inequity, appear to further consolidate this sorting logic. Some participants assumed, often without verification, that their admission chances would be lower outside their home zones. As one student (Bade, Male, Southwest) noted,

*"I wanted to study in another region, but I know I will not be a priority or might not get admission since I am not from the state, and they will give most slots to their indigenes. I knew I had no chance."*

Here, policy, perception, and precedent reinforce one another, leading students to pre-sort themselves before any formal selection occurs.

In effect, Primary Sorting produces a form of constrained mobility where proximity is overvalued not out of choice, but necessity. It reflects how the colonial logic of regional containment, where educational development followed uneven administrative lines, continues to shape access. As Mamdani (1996) suggests, decentralised despotism did not end with independence; it was inherited, naturalised, and extended. The educational landscape remains fragmented by history, and for rural students, movement across it is less a journey through opportunity than a negotiation with the limits of geography, memory, and structural precaution.

#### Secondary sorting: shared Identity, Spatial risks and the boundaries of belonging

Secondary Sorting refers to the identity-based filtering of university options among rural students. Unlike Primary Sorting, which is driven by proximity, this layer of decision-making concerns the anticipation of social legibility: the desire to enter environments where one’s language, religion, values, and appearance do not require constant defence or explanation. It also extends to how students weigh the risks of insecurity and the aspirations tied to positioning themselves in urban centres, producing a form of sorting that is as much about where one belongs as where one feels safe to pursue a future.

Students often select institutions where their own cultural codes will be immediately understood and unremarkable. This logic extends across all major regions. A Hausa-speaking student from the North, for example, may avoid Southern universities not because of geographic distance, but because of the dissonance anticipated in daily life.

*“Going to a university in the East or South? That’s difficult for me because I don’t speak Yoruba or Igbo. It would feel like starting life all over again*,*”* one participant (Danla, Male, Northwest) remarked.

For such students, staying within the northern region allows the maintenance of linguistic fluency, communal rhythm, and religious continuity.

Others expressed similar intentions around religion. A Muslim participant explained that she felt *“safer in a school where there are other Muslims*,*”* noting that it was not simply a matter of doctrine, but of everyday experience, how people eat, how they dress, how they mark time. Christian students articulated parallel concerns. One participant (Sade, Female, Southwest) recalled her parents advising her to study *“where Christianity is common*,*”* reflecting a protective impulse to shield young people from spaces perceived as religiously cacophonous or morally unfamiliar.

These decisions are often made with reference to older siblings, community members, or local elders. Many described how stories about cultural and insecurity discomforts in other regions, whether true, embellished, or cautionary, shaped their options before formal applications were even made.

*“My uncle told me stories of how hard it was studying in another region. He said*,* ‘Don’t go where you are not wanted.’ I took his advice*,*”* one student (Fati, Female, North central) recalled.

Similarly, a student added that,

*“Every day on the news*,* you hear of attacks*,* school closures… My parents said*,* ‘Why put yourself in danger when you can study somewhere safer?”* (Bade, Male, Southwest).

Another (Fati, Male, North West) noted,

*"It’s not even about discrimination, but some places have stereotypes about people from my tribe…they call us Aboki (derogatory way of calling Northerners), why put myself in that situation?"*

For others, they combined fear and aspiration.

*“I didn’t even consider applying to universities in the North. People from my area always talk about how it is not safe for us there. It’s not just about Boko Haram or security issues*,* it’s about feeling like you’re in a place that doesn’t welcome you.”* (Bola, Female, Southwest).

By contrast, some students pursued urban universities despite the cost and distance, framing the risk as necessary for future success.

*“People said I was crazy. No relatives there*,* and life is expensive… but I knew if I stayed in my town*,* even after graduation*,* I would still be struggling.”* (Sunmi, Male, South West).

In these accounts, identity is not a static descriptor but a mobility constraint. Students weigh whether their religious practices, clothing styles, or spoken language will mark them out as different. At the same time, they reckon with how insecurity or opportunity in particular regions might either intensify or ease those burdens. In contexts where the social environment is ambiguous or coded as hostile, many retreat to familiar zones. A female student from the East, for example, explained,

*I know I can survive in a university in the East because I am Igbo. If I go to the North*,* I will struggle with everything… I am studying at the University of Asa because I grew up here with my parents and speak Yoruba*.

Here, the university becomes not just a site of learning, but a space of identity regulation, where visibility is fraught, and difference is policed implicitly, if not explicitly.

Secondary Sorting therefore channels student mobility through familiar corridors. Institutions are not chosen solely on merit or location, but on their alignment with cultural assumptions. One student (Dami, Female, Southwest) captured this logic succinctly: *“I wanted to feel like I belong you know.”* The decision is not devoid of ambition, it is shaped by an intimate awareness of what it might mean to learn under constant surveillance or suspicion. This awareness is not imagined. It is cultivated through generational knowledge, reinforced through kinship networks, and intensified by ongoing regional tensions.

Secondary Sorting narrows choices in quiet but powerful ways. Keeping students within regions where their identity feels secure limits cross-regional contact and reinforces ethnic and religious divides. Universities end up more homogeneous, with diversity unevenly spread. For many rural students, this is less about rejecting opportunity than about balancing safety with the hope of a better future.

### Part II: institutional experiences of access

JAMB and Post JAMB and University Access.

For many rural students in Nigeria, accessing university education is not simply a matter of academic performance but a process of navigating the structural complexities of the JAMB system. Designed as a centralised gateway into university, JAMB, particularly since its shift to computer-based testing (CBT), has become a site where hoary inequities are reproduced rather than redressed.

Unlike their urban counterparts, rural students approach the examination from a position of disadvantage: limited access to digital infrastructure, scarce preparatory resources, and the financial strain of travelling long distances to exam centres. The transition from paper-based to CBT was intended to modernise assessment, yet for many, it has introduced new barriers to entry. As one student (Bada, Male, Northwest) shared:

*"The first time I wrote JAMB, I didn’t even know how to use the computer properly. I was just pressing anything because I had never practised before. They gave us a few minutes before the exam to try, but how does that help someone who has never even touched a mouse before? I was just clicking, dragging without knowing what it meant, and by the time the exam started, I was already panicking. I knew the answers in my head, but my hand and my eyes could not keep up with the computer. I walked out of that hall feeling as if the system was designed for people who already had the privilege of computers, not people like us."*

The digital divide means that rural students often require multiple attempts to achieve a qualifying score. For some, each failed attempt results not from a lack of intelligence but from unfamiliarity with the medium. One student recounted:

*“My laptop shut down in the middle of the exam. I do not know what happened; the screen just went black. I nearly had a heart attack in that hall. They later fixed it*,* but by then my mind was gone. I was shaking*,* sweating*,* and just clicking anyhow. I scored 103 in the JAMB that year*,* which was heartbreaking because I had prepared so hard. And yet*,* the result could not even be carried forward. I had to register and write another JAMB the following year*,* spending money again*,* repeating the stress again*,* and wasting another year of my life.”* (Tade, Female, North central).

Beyond these infrastructural barriers, participants repeatedly questioned the one-year validity of the JAMB result. For them, this policy magnified the injustice of the system. Passing once, they argued, should have been enough, yet the requirement to retake the exam if admission was not secured compounded exclusion. As one student explained:

*“Why is it that if you pass this year*,* the result cannot be used the next year? It’s like they are saying your effort expires after one year*,* no matter how good you were. I passed the first time with a high score*,* but because I didn’t get admission that year*,* I had to start all over again.* (Sade, Female, Southwest)

Another participant captured the discouragement more broadly:

*“I know friends who gave up after writing JAMB twice. Some of them scored very high the first time*,* but because the admission didn’t come*,* they had to repeat it. The second time*,* they didn’t perform as well*,* and that was the end. They just stopped trying. It kills your hope when you realise that even passing once is not enough*,* and each year you are forced to gamble again with your future. I almost gave up myself because after two tries*,* I started believing maybe university was not for people like us.”* (Bade, Male, Southwest).

For those who manage to clear JAMB, the next obstacle is Post-JAMB screening, often referred to as Post-UTME. Students described this stage as another layer of exclusion, one that particularly disadvantages those from rural areas who cannot afford repeated travel or additional fees. As one participant explained:

*“After all the struggle of JAMB*,* we still had to pay again for Post-UTME. The exam was on the computer*,* and I had never used that type of software before. The questions kept moving*,* the time was running*,* and I could not even go back to check the ones I had skipped. By the time I know how the thing works and click properly*,* like 5 minutes is already gone…” (Hassan*,* Male*,* North West)*.

Another student highlighted how universities’ discretionary control over Post-UTME undermined the sense of fairness supposedly guaranteed by JAMB:

*“In JAMB I scored 200*,* which was good. But during Post-UTME*,* they used an exam I didn’t understand and then combined it with my JAMB score in a way I still don’t know. They never explained how the final marks were calculated. Me I think I failed to get admission that year not because I wasn’t qualified*,* but because the system was not transparent. For me*,* Post-UTME feels like a way to cut people out.” (Dami*,* Female*,* Southwest)*.

These testimonies reveal how a policy framed as standardisation produces unintended barriers that discourage persistence, particularly among rural youth who already bear cumulative disadvantages. What emerges is not just exclusion at the point of entry, but a cycle of discouragement where the denial of result validity thwarts opportunity and weakens resilience.

Logistics compounds this challenge. Exam centres are often located in urban areas, far from rural communities. The financial and emotional toll of travelling to unfamiliar cities just to take an exam adds further strain:

*“My JAMB centre was in a city far from my village. I had to travel the day before to sleep with brother Wasiu and wake up early to find the venue… by the time the exam started*,* my brain was already tired. (*Bada, Male, Northwest)

Although JAMB is often upheld as a meritocratic model of fair selection and response to curb exam malpractice, students’ testimonies expose it as a system that disproportionately penalises those without digital access, financial security, or proximity to support networks. As one participant put it:

*“People say if you fail JAMB*,* you are not serious. But they don’t see what we go through. Me I know It’s not just about intelligence*,* it’s about where you are coming from… (*Dami, Female, Southwest).

Taken together, these accounts show that access to university education in Nigeria is stratified not only by performance but also by geography, infrastructure, bureaucratic competence, and the rigid rules governing JAMB’s single-year validity.

## Discussion and conclusion

The findings of this study reveal that access to university in rural Nigeria is not a discrete moment of admission, but an ongoing process shaped by academic progression, spatial negotiation, and historical reckoning. Rather than aligning with policy imaginaries of meritocratic inclusion, students’ narratives unveil what Massey (1993) might call a power geometry, a geography of constraint in which routes, risks, and refusals are structured by inherited spatial scripts, infrastructural absences, and institutional indifference. The university choice logics, staying close to home (primary sorting), shared identity, spatial risks and the boundaries of belonging (secondary sorting) and navigating the barriers of JAMB and post-JAMB, demonstrate that students do not choose institutions freely but rather move within what Bourdieu (1977) terms fields of possibility, where habitus conditions what feels attainable and where.

This spatial logic, one could argue, is neither new nor accidental. Mamdani’s (1996) theorisation of decentralised despotism is particularly instructive in reading how colonial spatial divisions, rooted in indirect rule and selective missionary investment, have sedimented into enduring regional hierarchies of access. The persistent divide between Nigeria’s North, South, and East is not only infrastructural or administrative but symbolic and affective, manifesting in students’ anxieties about belonging, safety, and institutional recognition. When a male student from the Southwest spoke of avoiding universities in the North because “people from my area always talk about how it is not safe for us there,” or when a Northern student recalled being labelled “Aboki” in the South, these are not individual fears alone. They are performances of spatial politics, ways in which colonial and postcolonial histories of suspicion, violence, and uneven development are kept alive through everyday choices of where to go and where to avoid. Avoidance, as shown in this study, is not irrational conservatism but what Evans (2002) describe as “bounded agency”, strategic choices made under structural constraint and interpreted through generational memory.

Furthermore, this is where the politics of space becomes visible. Students’ decisions reproduce and reinforce invisible borders that map onto Nigeria’s ethno-regional divides, thereby shaping the very geography of HE. Choosing one’s state, one could argue, is about a personal act of safety and a political act of containment, visibilising colonial logics of decentralised governance. Likewise, the post-JAMB and JAMB systems, with their digital exclusions, annual validity rules, and urban-centred exam centres, are not neutral mechanisms but a political technology of sorting, one that privileges some regions and student populations over others. Bourdieu’s theoretical logic of habitus helps to explain how these structural asymmetries are lived and internalised. For rural students, habitus mediates the tension between aspiration and constraint: it frames proximity as safety, unfamiliar regions as risk, computer-based exams as sites of anxiety, and certain institutions as out of reach even before applications are made. Educational choices are thus not merely strategic calculations but embodied responses to inherited geographies and technological exclusions. This reinforces the argument that university access in postcolonial contexts must be situated within broader frameworks of spatial justice (Soja, 2010; Donnelly & Evans, 2016). Where students choose to go, and perhaps more revealingly, where they refuse to go, is shaped not only by institutional reputation or affordability, but by historically sedimented readings of risk and familiarity. These readings are relational: regional identity, religious background, and linguistic fluency operate as markers of self and as tools for navigating terrain where universities signal different degrees of legibility and threat. As Power and Frandji (2010) argue, what is often misread as autonomous preference is, in fact, strategic conformity, deeply structured by spatial and social constraint.

Moreover, these findings invite a rethinking of how aspiration is conceptualised in rural contexts. Dominant discourses often equate upward mobility with spatial movement, framing students who stay within their zones as lacking ambition or imagination. However, this study challenges such framings. For many participants, remaining close to home was not a failure of vision but a form of spatial care, an ethics of caution rooted in economic precarity, familial obligation, and the affective cost of unfamiliar terrain. Aspiration, then, is not always expansive; it can also be conservative, careful, and deeply informed by the risks of displacement and the fear of institutional alienation. This corroborates with Wolfe’s (2023) study in Australia, where rural aspirations were shaped by affective geographies of belonging, discomfort, and hope. In Nigeria too, staying close reflects affective as well as material constraints, as fear of displacement and search for safety structure the horizon of possibility. Aspiration is thus relational, anchored in histories of exclusion and the embodied pursuit of recognition.

Furthermore, this resonates with rural and minority ethnic HE researches in other settings, such as the UK and Australia, where studies have similarly challenged the pathologisation of staying close to home as ‘lacking aspiration’ (Corbett, 2000; Reay et al., 2005; Cuervo & Wyn, 2014). Corbett’s (2000) work on Canadian coastal communities, for example, centres how staying put can be a rational and emotionally grounded choice rather than a deficit. Likewise, in South Africa, Walker and Mathebula (2020) show that young people’s decisions to attend local institutions are shaped by unequal access to information, perceived institutional hostility, and socio-economic barriers. These comparative insights highlight the need to read rural educational trajectories not as anomalies but as valid adaptations to uneven geographies of opportunity.

Moreover, unlike some Global South contexts where rural marginality is tied predominantly to economic deprivation, in Nigeria, it is also entangled with ethno-religious stratification and geopolitical legacies of colonial rule, making rurality not just a matter of location, but a site of layered governance, historical neglect, and epistemic devaluation (Olaniyan, 2025). Access here is deeply political because it is about who has the right to cross borders, who is rendered out of place, and how state policies and institutional practices silently reinscribe colonial-era geographies under the guise of neutrality.What this study contributes, then, is a contextualised understanding of how rurality, in the Nigerian context, functions not just as a geographic descriptor but as a structuring force of institutional legibility, access, and legitimacy. It also expands the conceptual grammar of access and invites comparative work on postcolonial HE systems where spatial and historical legacies persist in shaping who gets to be seen, heard, and supported. To move forward, access must be understood as project that dismantles inherited inequalities and creates conditions where rural students can pursue futures without negotiating safety, belonging, and recognition at every step.

## Data Availability

Anonymized data can be provided upon request, subject to ethical approval.
